# Effects of exercise and doxorubicin on acute diaphragm neuromuscular transmission failure

**DOI:** 10.1016/j.expneurol.2024.114818

**Published:** 2024-05-21

**Authors:** Branden L. Nguyen, Dryden R. Baumfalk, Stephanie S. Lapierre-Nguyen, Renjia Zhong, Vivian Doerr, Ryan N. Montalvo, Lan Wei-LaPierre, Ashley J. Smuder

**Affiliations:** Department Applied Physiology and Kinesiology, University of Florida, 1864 Stadium Rd., Gainesville, FL 32611, United States of America

**Keywords:** Respiratory muscles, Chemotherapy, Neuropathy, Phrenic nerve, Exercise

## Abstract

Doxorubicin (DOX) is a highly effective anthracycline antibiotic used to treat a wide variety of cancers including breast cancer, leukemia and lymphoma. Unfortunately, clinical use of DOX is limited due to adverse off-target effects resulting in fatigue, respiratory muscle weakness and dyspnea. The diaphragm is the primary muscle of inspiration and respiratory insufficiency is likely the result of both muscle weakness and neural impairment. However, the contribution of neuropathology to DOX-induced respiratory muscle dysfunction is unclear. We hypothesized that diaphragm weakness following acute DOX exposure is associated with neurotoxicity and that exercise preconditioning is sufficient to improve diaphragm muscle contractility by maintaining neuromuscular integrity. Adult female Sprague-Dawley rats were randomized into four experimental groups: 1) sedentary-saline, 2) sedentary-DOX, 3) exercise-saline or 4) exercise-DOX. Endurance exercise preconditioning consisted of treadmill running for 1 h/day at 30 m/min for 10 days. Twenty-four hours after the last bout of exercise, animals were treated with DOX (20 mg/kg, I.P.) or saline (equal volume). Our results demonstrate that 48-h following DOX administration diaphragm muscle specific force is reduced in sedentary-DOX rats in response to both phrenic nerve and direct diaphragm stimulation. Importantly, endurance exercise preconditioning in DOX-treated rats attenuated the decrease in diaphragm contractile function, reduced neuromuscular transmission failure and altered phrenic nerve morphology. These changes were associated with an exercise-induced reduction in circulating biomarkers of inflammation, nerve injury and reformation. Therefore, the results are consistent with exercise preconditioning as an effective way of reducing respiratory impairment via preservation of phrenic-diaphragm neuromuscular conduction.

## 1. Introduction

Doxorubicin (DOX) is a member of the anthracycline family of chemotherapeutic agents used to treat a variety of solid tumor, gynecological and blood cancers (Carter, 1975). Although highly effective at reducing tumor burden, a common side effect of DOX includes severe respiratory muscle weakness (Travers et al., 1985; Stevens and Sporn, 2014; Gilliam et al., 2011a). Respiratory insufficiency leads to dyspnea, decreases in maximal inspiratory and expiratory pressures and exercise intolerance, which limits patient performance of activities of daily living, reduces quality of life and increases the risk of co-morbidities (Travers et al., 1985; Stevens and Sporn, 2014; Schwartz et al., 2007; Elbl et al., 2006; Norton et al., 2020). As the mechanisms responsible for DOX-induced muscle weakness continue to be elucidated, emerging evidence suggests that neuropathology may be a contributing factor (Bonifati et al., 2000; Nyrop et al., 2019; Bandos et al., 2018; Rivera et al., 2018). Thus, determining the contribution of neurotoxicity to DOX-induced respiratory muscle weakness is clinically important to identify potential therapeutic targets to improve patient quality of life and survival.

Previous work investigating respiratory dysfunction following acute DOX exposure has primarily focused on muscle-specific reductions in contractility and fiber size (Min et al., 2015; Morton et al., 2019; Gilliam et al., 2011b; Doerr et al., 2022). However, in vitro evidence from multiple neuronal populations (Zhou et al., 2018; Shokoohinia et al., 2014; Alhowail et al., 2019) and in vivo assessment of the hypercapnic chemoreflex (Morton et al., 2019) support the postulate that DOX neuropathology contributes to respiratory insufficiency. Specifically, the blunted ability to increase both respiratory rate and tidal volume in response to a hypercapnic challenge suggests impairments to rhythm-generating circuits, chemosensation and/or diaphragm weakness (Fields et al., 2019). In contrast, rats that have undergone exercise preconditioning prior to DOX treatment retain full capacity to increase ventilation during a hypercapnic challenge and demonstrate preserved diaphragm muscle force generating capacity (Morton et al., 2019).

The goal of this study was to advance our understanding of the factors mediating respiratory deficiency following DOX treatment and further clarify the protective effects of exercise. To this end, comparison of phrenic nerve evoked diaphragm force to direct muscle stimulation revealed comparable deficits with acute DOX treatment and improvements with exercise preconditioning. However, exercise was sufficient to attenuate DOX-induced neuromuscular transmission failure (NMTF). This improvement in communication between the pre-synaptic axon terminals and post-synaptic motor endplates was not associated with altered neuromuscular junction (NMJ) morphology but was potentially related to improved phrenic nerve conduction and reduced neural inflammation. These results provide new and important insights concerning the pathogenesis of DOX-induced respiratory impairment and the beneficial effects of exercise to combat these effects.

## 2. Methods

### 2.1. Experimental groups

All animal experiments were approved by the University of Florida Institutional Animal Care and Use Committee. Adult female Sprague-Dawley rats (4–6 months old; Charles River Laboratories, Wilmington, MA) were randomly assigned to the following experimental groups: 1) sedentary-saline (Sed-Sal) (*n* = 10), 2) sedentary-doxorubicin (Sed-DOX) (*n* = 9), 3) exercise-saline (Ex-Sal) (*n* = 10), and 4) exercise-doxorubicin (Ex-DOX) (n = 10). While DOX myotoxicity is established in both male and female rodents (Montalvo et al., 2021; Podyacheva et al., 2021), female rats were utilized in this study due to the extensive and effective use of DOX to treat gynecological cancers (Guigni et al., 2018; Siegel et al., 2023; Rahaman et al., 2009). Although, hormonal changes occur in females affected by gynecological and breast cancers, sex hormone production was not modified to eliminate potential confounding effects of hormone deficiency on diaphragm function (Vang et al., 2021). Animals were housed in the University of Florida Animal Care facility on a 12:12-h light-dark cycle and were provided food and water ad libitum.

### 2.2. Endurance exercise treadmill training

Preconditioning exercise is both an important tool to elucidate potential targets for therapeutic intervention (Powers et al., 2018; Smuder, 2019) and a potential clinical intervention to prevent DOX toxicity (Kirkham et al., 2017). Animals assigned to the exercise trained groups completed progressive habituation to treadmill running for 5 consecutive days (10, 20, 30, 40, and 50 min/day at 30 m/min, 0% grade on days 1–5, respectively). Following two days of rest, animals began treadmill running (1 h/day at 30 m/min, 0% grade) 5 days/week for two weeks (Lawler et al., 1993). Importantly, this exercise preconditioning protocol has been shown to result in exercise-induced adaptations that protect against DOX-induced diaphragm muscle weakness (Morton et al., 2019). All animals completed this protocol in its entirety and no animals were removed due to inability to run. Sedentary animals were exposed to the non-moving treadmill. Twenty-four hours following the last bout of exercise, animals were treated with saline or DOX.

### 2.3. Doxorubicin treatment

Animals assigned to DOX treated groups received an intraperitoneal injection (I.P.) of doxorubicin hydrochloride, pH 7.0 (20 mg/kg body weight, Teva Pharmaceuticals, Parsippany, NJ) forty-eight hours prior to sacrifice. This dose is equivalent to a clinically prescribed dose of DOX for cancer treatment (Hiensch et al., 2020; Johnson-Arbor and Dubey, 2024) and is established to cause diaphragm muscle toxicity in rodent models (Gilliam et al., 2011a; Morton et al., 2019; Gilliam et al., 2011b). Animals in the saline treated groups received an I.P. injection of an equal volume forty-eight hours prior to sacrifice.

### 2.4. Tissue harvest

Forty-eight hours following DOX or saline treatment, rats were anesthetized via inhaled isoflurane. After reaching a surgical plane of anesthesia, the left phrenic nerve was carefully isolated and remained innervated to the left hemidiaphragm for in vitro functional experiments. The right phrenic nerve proximal to the diaphragm muscle fibers (~3–4 mm) was removed and prepared for morphological analysis. Remaining diaphragm muscle tissue was frozen in liquid nitrogen and stored at − 80 °C.

### 2.5. In vitro phrenic nerve and diaphragm muscle force production

A muscle strip containing the central tendon, rib and intact phrenic nerve was suspended vertically between two platinum electrodes with the tendon connected to an isotonic force transducer (Aurora Scientific, Aurora, ON, Canada) within a jacketed tissue bath at 25 °C containing Krebs-Henseleit buffer equilibrated with 95% oxygen and 5% carbon dioxide gas. Within the bath, the phrenic nerve was attached to a suction electrode (A-M Systems, Carlsborg, WA) to stimulate phrenic nerve-dependent diaphragm force production. Following equilibration, optimal length was determined by supramaximal twitch stimulations (1 Hz) by the flanking platinum electrodes (model FT-03, Grass Instruments, Quincy, MA) at increasing length until peak active force was determined. When optimal length was found, the phrenic nerve was stimulated via the suction electrode to determine the optimal amperes (mA) for a peak active force. Once optimal mA was determined, a force-frequency response through phrenic nerve stimulation was determined in the diaphragm muscle strip by stimulating the phrenic nerve at 15, 30, 60, 100 and 160 Hz (Aurora Scientific, Aurora, ON, Canada, 120 V, 0.25 ms pulses). Five minutes following the completion of the phrenic nerve stimulated force-frequency response, the direct diaphragm muscle force-frequency response was measured by utilizing the flanking platinum electrode with supramaximal stimulations (120 V, 15–160 Hz, 600 mA, 0.25 ms, model FT-03, Grass Instruments, Quincy, MA). All forces were normalized to cross-sectional area as previously described (Segal et al., 1986). If the phrenic nerve was damaged during dissection the animal was removed from all diaphragm force analyses.

### 2.6. Assessment of diaphragm neuromuscular transmission failure

To determine diaphragm NMTF, the following fatiguing protocol was adapted from previous reports (Fogarty et al., 2019; Khurram et al., 2018; Fogarty et al., 1985). Briefly, the phrenic nerve was stimulated via the suction electrode at 30 Hz every 2 s (s) for 180 s. At the 30 s, 60 s, 90 s, 120 s, 150 s, and 180 s timepoint, the suction electrode stimulator was shut off to prevent phrenic nerve-induced force production and the diaphragm muscle was stimulated via the flanking platinum electrodes (Grass Stimulator, 30 Hz, 0.25 ms pulse, 600 mA). When muscle fibers are not activated by repetitive nerve stimulations, these muscle fibers are not affected by muscle-derived fatigue (Rowley et al., 2005; Rowley et al., 2007). Therefore, with NMTF, the difference between forces generated by nerve stimulation compared to direct muscle stimulation is greater. The percentage of NMTF is an indication of the reduction in force related to neuromuscular transmission and not related to diaphragm muscle fatigue (Fogarty et al., 2019; Fogarty et al., 1985; Kuei et al., 1985; Sieck and Prakash, 1995). Briefly, the initial diaphragm forces evoked by phrenic nerve stimulation (${NF}_{\text{init}}$) and direct muscle stimulation $\left({MF}_{\text{init}}\right)$ were measured at the beginning of the neuromuscular transmission fatiguing protocol. NMTF was calculated using the following equation as previously described (Fogarty et al., 2019; Khurram et al., 2018; Fogarty et al., 1985):

(1) $$NMTF=\left[MF/{MF}_{\text{init}}NF/{NF}_{\text{init}}\right]/\left[MF/{MF}_{\text{init}}\right]\times 100$$

### 2.7. Phrenic nerve morphology

To examine nerve morphology by bright-field microscopy, phrenic nerves were fixed in 4% paraformaldehyde and 2.5% glutaraldehyde in 0.1 M cacodylate buffer containing 2 mM MgCl_2_, 1 mM CaCl_2_ and 0.25% NaCl (pH: 7.24) for one hour at room temperature. Fixed tissues were processed at the University of Florida Interdisciplinary Center of Biotechnology Research Electron Microscopy Core. Samples were buffer washed and encapsulated in buffered 3% low-temperature gel agarose. Specimens were post-fixed with buffered 1% osmium tetroxide, buffer washed, water washed and dehydrated in an ascending series of graded ethanol of 25% to 100% in 5–10% increments, followed by 100% anhydrous acetone. Dehydrated samples were infiltrated in graded acetone-Embed/Araldite epoxy resin with Z6040 embedding primer (Electron Microscopy Sciences, Hatfield, PA) at 30%, 50%, 70% and 100%, then cured at 70 °C. Semi-thick sections (500 nm) were collected and stained with toluidine blue for 10 min at 50 °C. Glass coverslips were mounted onto the slides with Eukitt Mounting Medium (Electron Microscopy Sciences, Hatfield, PA). Phrenic nerve fiber diameter (axon with myelin, 500–600 fibers/animal) and myelin sheath thickness (500–600 fibers/animal) were measured. Phrenic nerve g-ratio was determined by calculating both the outer diameter of myelinated axons and the inner membrane representing the axonal diameter alone as previously described (Hiensch et al., 2020). Data was analyzed using public domain NIH Image J software (Falk et al., 2015).

### 2.8. Confocal immunofluorescent microscopy and analysis of diaphragm NMJ morphology

Muscle bundles from the mid-costal diaphragm were used to determine NMJ morphology. Fascia was first removed followed by fixation in 3.2% paraformaldehyde for 10 min and washed in cold PBS. The diaphragm bundles were then placed in blocking solution consisting of 1% Triton X-100 and 4% BSA in PBS at 4 °C for 24 h. After incubating in blocking solution, diaphragm muscle bundles were placed in primary antibody cocktail containing anti-synaptotagmin/ZNP-1 (1:200, Zebrafish International Resource Center) and anti-neurofilament (NFH, 1:5000, EncorBio, CPCA-NF-H) at 4 °C for 24–48 h. Following primary antibody incubation muscle bundles were placed in a secondary antibody cocktail containing anti-mouse Alexa 488 (1:250, Jackson ImmunoLabs, 715–545–150), anti-chicken Alexa 647 (1:1000, Life Technologies, A21449) and Alexa 594 Bungarotoxin (1:1000, Life Technologies, B13423) in blocking solution for 16–20 h at 4 °C. All slides were mounted with Prolong Gold with anti-fade containing DAPI (Life Technologies, P36931) and stored at 4 °C for subsequent analysis. Muscle bundles were imaged on a Leica Confocal Microscope. Diaphragm bundles were viewed under 40× magnification and 2.5 zoom. Leica Application Suite × software was used to generate 3-D reconstructed NMJs for morphology. NMJ morphology was determined as previously described (Liu et al., 2017). Due to the subjective feature of this analysis, the 3-D images were analyzed separately by two investigators blinded to treatments to avoid inter-personal bias. Briefly, an NMJ was considered to be: 1) innervated, if >50% of the post-synaptic area was covered by pre-synaptic terminal markers; 2) partially innervated, if 10–50% length of an acetylcholine receptor (AChR) branch within the post-synaptic area is not covered by pre-synaptic terminal markers or 3) degenerated, if the AChR-enriched area resembles a patch devoid of defined elaborate branches with <10% overlap (Liu et al., 2017; Zhang et al., 2007). In addition, NMJ morphology was categorized as plaque-like to a pretzel-like shape to assess complexity of post-synaptic structure, while fragmentation of NMJs were categorized into 1, 2, 3 or 4 or more fragments by counting the number of islands in the post-synaptic area (Liu et al., 2017; Liu et al., 2015).

### 2.9. Luminex analysis of plasma myokines

Approximately 3 mL of blood was collected via cardiac puncture into K_3_ EDTA coated blood collection tubes. Collection tubes were then centrifuged at 5000 rpm for 10 min at 4 °C. Immediately following, the plasma was collected and stored at − 80 °C for subsequent analysis. Brain-derived neurotrophic factor (BDNF), erythropoietin (EPO), follistatin-1 (FSTL-1), fractalkine, myostatin (MSTN/GDF8), osteocrin, secreted protein acidic and rich in cysteine (SPARC) and fibroblast growth factor 21 (FGF21) were measured using MILLIPLEX^®^ MAP Rat Myokine Immunology Multiplex assays on a Luminex MAGPIX detection system. Standard curves of each analyte were visualized and optimized to the best fit (either 4- or 5- parameter logistic method) using Millipore Belysa^®^ Immunoassay Curve Fitting Software (Merck KGaA, Darmstadt, Germany). Non-determined values and those that fell below the value of the first standard were designated as below the level of quantification and were removed from analysis.

### 2.10. Statistical analysis

Statistical analysis was performed using GraphPad Prism 9 (GraphPad Software, La Jolla, CA). Normality was tested by Shapiro-Wilk for all data sets. A two-way ANOVA was used to determine main effects of activity (Sed/Ex), treatment (Sal/DOX) and interaction (activity × treatment). When appropriate Tukey’s multiple comparisons test was used post hoc to determine differences between groups. A Kruskal-Wallis test and Dunn’s post-hoc test was used to compare dependent measures for phrenic nerve morphology. Significance was established at *P* < *0.05*. Data are presented as means ± standard error (SE).

## 3. Results

### 3.1. Body weight change

No differences existed in body weight between groups prior to Sal/DOX treatment or at the experimental endpoint (data not shown). Comparison of body weight change (endpoint – treatment) revealed a significant main effect of DOX treatment and activity (*P* < *0.05*). Specifically, Sed-DOX rats lost significantly more weight compared to Sed-Sal and Ex-Sal rats (*P* < *0.05*, Table 1). However, Ex-DOX rats lost significantly more weight compared to Ex-Sal rats, with no significant differences in weight loss between Sed-Sal and Sed-DOX (*P* < *0.05*, Table 1).

### 3.2. Exercise prevents DOX-induced diaphragm muscle contractile dysfunction

**Fig. 1.**
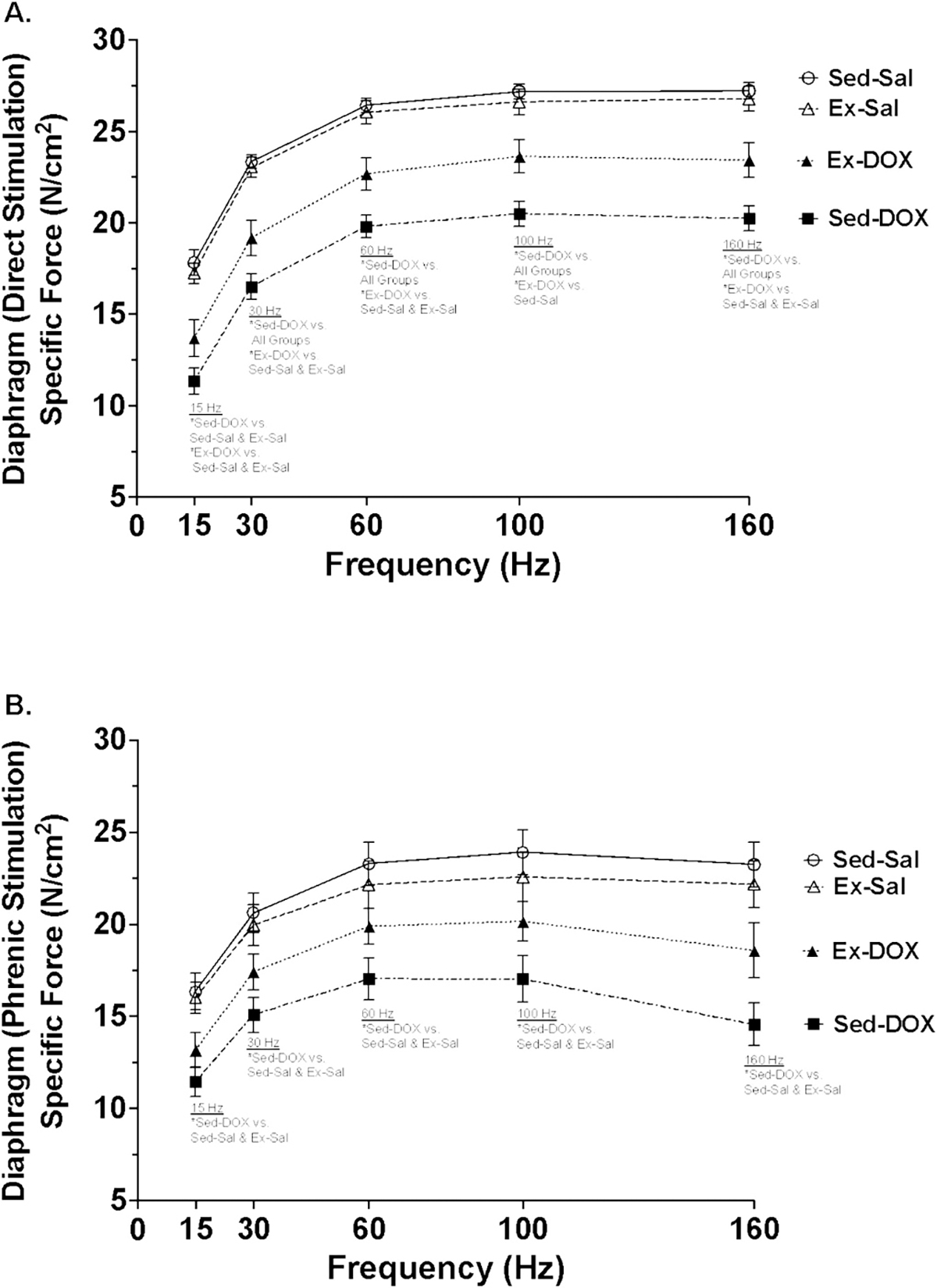
(A-B). Ex vivo diaphragm muscle specific force (A-B). A) Direct diaphragm muscle stimulation force-frequency curve. B) Phrenic nerve-stimulated diaphragm muscle force-frequency curve. A two-way ANOVA and Tukey’s multiple comparisons test was used. Group differences are shown in text on the graphs (*P* < *0.05*). *n* = 8/group. Sample size was modified based on the removal of animals whose phrenic nerve was damaged during dissection. Graphs represent mean ± SE.

Respiratory muscle weakness is a major contributor to dyspnea and exercise intolerance in patients treated with DOX (Elbl et al., 2006). Therefore, we evaluated the effect of exercise preconditioning on diaphragm muscle contractility. Evaluation of the diaphragm muscle force-frequency response, independent from phrenic nerve-induced force production showed a significant interaction at 30, 60, 100 and 160 Hz, with a main effect of DOX treatment for all frequencies tested (*P* < *0.05*; 15–160 Hz) (Fig. 1A). Specifically, at 15 Hz both Sed-DOX and Ex-DOX produced less force compared to Sed-Sal and Ex-Sal (*P* < *0.05*). At 30–160 Hz Sed-DOX produced lower force compared to all other groups (*P* < *0.05*). Ex-DOX had lower specific force production compared to both Sed-Sal and Ex-Sal at 30, 60 and 160 Hz, but was only significantly weaker than Sed-Sal at 100 Hz (*P* < *0.05*). Similar results were shown when the phrenic nerve was stimulated to determine diaphragm muscle force production (Fig. 1B). Evaluation of the phrenic nerve-evoked force frequency response demonstrated a significant main effect of DOX treatment throughout the frequencies tested (*P* < *0.05*). Multiple comparisons showed a significant reduction in diaphragm muscle force generation in the Sed-DOX group compared to Sed-Sal and Ex-Sal for each frequency when stimulated by the phrenic nerve (*P* < *0.05*).

### 3.3. Exercise preconditioning prevents DOX-induced diaphragm neuromuscular transmission failure

**Fig. 2.**
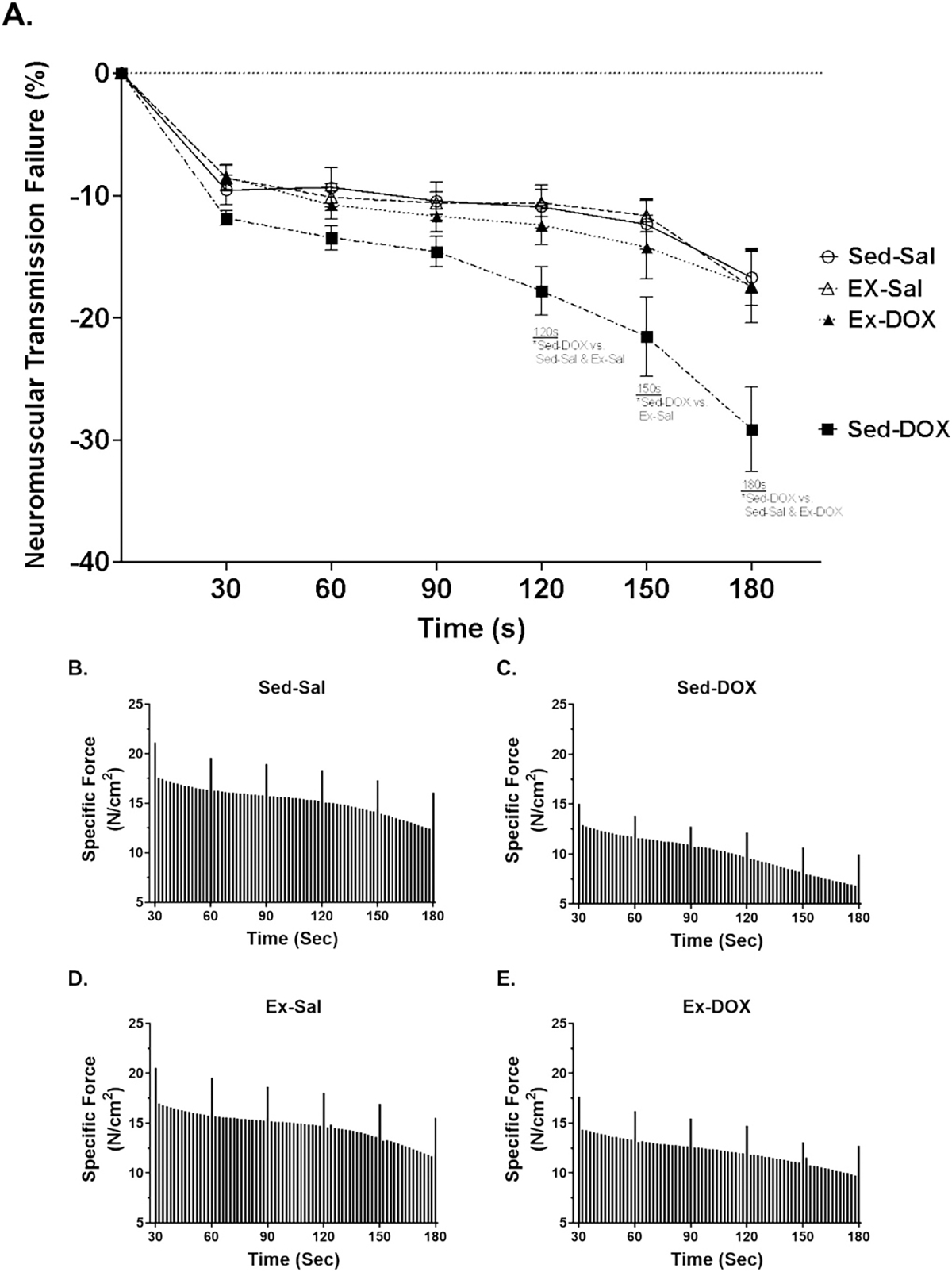
(A-E). Diaphragm Neuromuscular Transmission Failure (NMTF). A) NMTF was determined in the diaphragm by repetitive 30 Hz stimulations. Representative traces shown in B) Sed-Sal; C) Sed-DOX; D) Ex-Sal and; E) Ex-DOX. Main effects and interaction were determined by two-way ANOVA and a Tukey’s multiple comparisons indicated by text on the graph (*P* < *0.05*). *n* = 8/group. Samples size was modified based on the removal of animals whose phrenic nerve was damaged during dissection. Graphs represent mean ± SE.

We further evaluated the effects of DOX treatment on neuromuscular function by determining diaphragm NMTF during a fatiguing protocol (Fig. 2A–E). At the 30 s timepoint, there was no difference in NMTF between groups; however, there was a main effect of activity to reduce NMTF (*P* < *0.05*, Fig. 2E). There were no differences at the 60 s and 90 s timepoint. At the 120 s timepoint there was a main effect of treatment, with the percent NMTF for Sed-DOX rats significantly greater compared to Sed-Sal and Ex-Sal (*P* < *0.05*, Fig. 2E). Our results also demonstrate a main effect of treatment at the 150 s stimulation and an increase in NMTF in Sed-DOX compared to Ex-Sal (*P* < *0.05*, Fig. 2E). Finally, at 180 s our analysis showed an interaction and a main effect of treatment (*P* < *0.05*, Fig. 2E). Post hoc analysis revealed NMTF was greater in Sed-DOX compared to Sed-Sal and Ex-DOX (*P* < *0.05*, Fig. 2E).

### 3.4. Exercise preconditioning prevents DOX-induced phrenic nerve injury

**Fig. 3.**
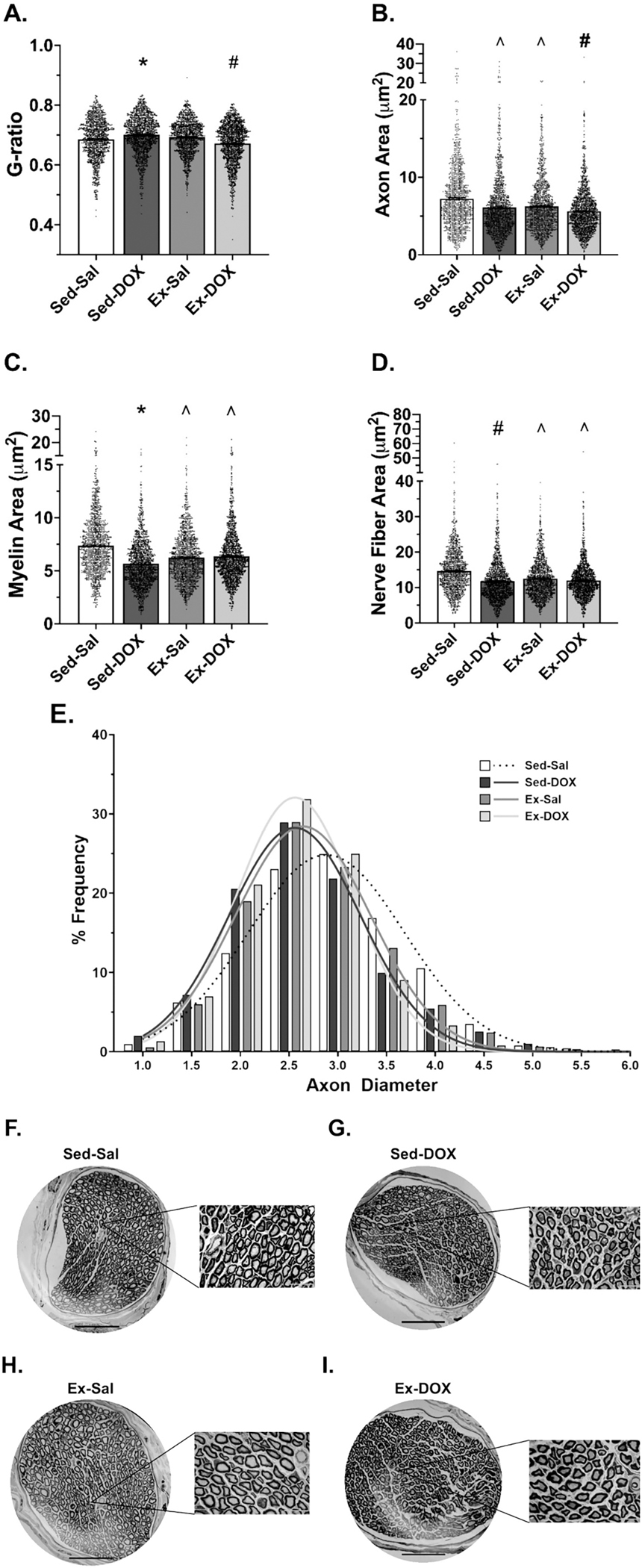
(A-I). Phrenic Nerve Morphology. Phrenic nerve cross-sections were analyzed (500–600 nerve fibers/animal; *n* = 4–5/group) for A) g-ratio; B) axon area; C) myelin area; D) nerve fiber area and; E) axon diameter distribution. Representative images shown in F) Sed-Sal; G) Sed-DOX; H) Ex-Sal and; I) Ex-DOX. Statistical analysis was completed by Kruskal-Wallis test and Dunn’s multiple comparisons test. * = significantly different vs all groups. # = significantly different vs Sed-Sal and Ex-Sal. ^ = significantly different vs Sed-Sal. Graphs represent mean ± SE. Black circles depict individual nerve fibers. Black scale bar represents 30 μm. White scale bar represents 20 μm.

Morphometric analysis of phrenic nerve cross-sections was performed to assess markers of axonal function and integrity (Cercignani et al., 2017). G-ratio was calculated as the ratio of the inner (axon only) and total diameter (axon + myelin) of the nerve fiber (Fig. 3A–I). Our results show that the g-ratio for Sed-DOX rats was greater compared to all other groups, with Ex-Sal and Sed-DOX also greater than Ex-DOX (*P* < *0.05*, Fig. 3A). Axon area was smaller in Sed-DOX and Ex-Sal rats compared to Sed-Sal, and smaller in Ex-DOX compared to Sed-Sal and Ex-Sal (*P* < *0.05*, Fig. 3B). Similarly, the frequency distribution of phrenic axon diameter was shifted leftward in Sed-DOX rats compared to Sed-Sal (Fig. 3E). Myelin area was reduced in Sed-DOX rats compared to all groups and also in Ex-Sal and Ex-DOX rats compared to Sed-Sal (*P* < *0.05*, Fig. 3C). Correspondingly, total nerve fiber area was lower in Sed-DOX rats compared to Sed-Sal and Ex-Sal, with total area also less in Ex-Sal and Ex-DOX rats compared to Sed-Sal (*P* < *0.05*, Fig. 3D).

### 3.5. Diaphragm NMJ morphology

**Fig. 5.**
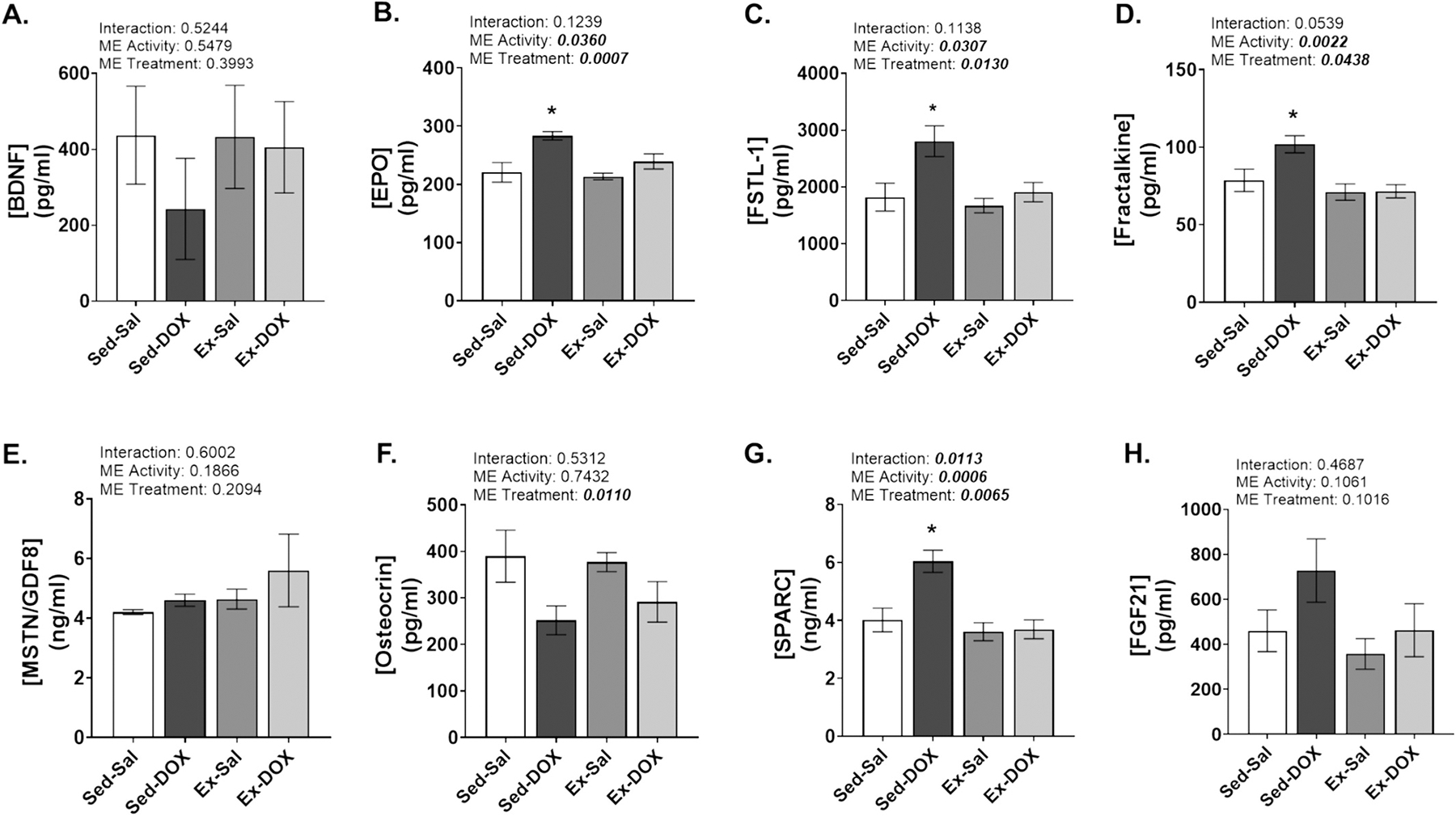
(A-H). Circulating plasma chemokine assay (*n*=6–10/group). Plasma levels of A) BDNF; B) EPO; C) FSTL-1; D) Fractalkine; E) MSTN/GDF8; F) Osteocrin; G) SPARC; and H) FGF21 were determined by multiplex assay. Statistical analysis was completed by two-way ANOVA and Tukey’s multiple comparisons test. Data are presented as mean ± SE. Significant main effects (ME) of activity, treatment or interaction are indicated in bold and italicized (*P* < *0.05*). * = significantly different vs all groups (*P* < *0.05*).

Confocal imaging of phrenic-diaphragm NMJ innervation (Fig. 5A), post-synaptic morphology (Fig. 5B) and post-synaptic degeneration (Fig. 5C) showed no differences between groups. However, there was a main effect of exercise to decrease the number of NMJs with 1 fragment and increase the number of NMJs with 2 fragments compared to sedentary groups (*P* < *0.05*, Fig. 5D).

### 3.6. Plasma myokines

Plasma was collected to determine the effect of exercise preconditioning and DOX treatment on circulating myokines (Fig. 5A–H). Our results demonstrate no significant change in circulating BDNF, MSTN/GDF8, Osteocrin or FGF21. Plasma levels of EPO, FSTL-1, Fractalkine and SPARC were significantly increased in the Sed-DOX group compared to all other groups (*P* < *0.05*).

## 4. Discussion

DOX impairs diaphragm muscle force production and promotes fatigue (Gilliam et al., 2011a; Doerr et al., 2022). However, these functional deficits can precede the onset of muscle atrophy and thus may be associated with neural pathology (Deschenes et al., 2010). In this regard, neuropathy is a dose-limiting toxicity associated with DOX chemotherapy treatment and can manifest as motor impairment, muscle weakness and fatigue (Kaminska and Cudnoch-Jedrzejewska, 2023). While previous studies have largely overlooked neural impairment as a contributing factor to DOX-induced muscle weakness, here we identify that NMTF, morphological alterations to phrenic axons and elevated plasma chemokines occur in conjunction with reduced diaphragm muscle contractile function. In contrast, physical activity is established to attenuate the adverse effects of DOX on diaphragm muscle function and to improve respiratory fitness and capacity (Morton et al., 2019; McNeely et al., 2006; Pinto and Maruyama, 1999; Montalvo et al., 2023). Although reports have associated the beneficial effects of exercise to reduce DOX myotoxicity with improved mitochondrial function and intracellular calcium handling (Morton et al., 2019; Powers et al., 2018; Smuder, 2019; Park et al., 2019; Marques-Aleixo et al., 2015; Smuder et al., 1985a), physical exercise can also elicit neuroplasticity by increasing quantal content, the size of the neuromuscular synapse and myelination to enhance neuromuscular transmission (Desaulniers et al., 1985; Cheng et al., 2020). Thus, the protective effects of exercise to reduce diaphragm muscle weakness and fatigue appears to be associated with improved neuromuscular communication. We conclude that neural control of the diaphragm is impaired following acute DOX treatment and that exercise preconditioning prevents diaphragm NMTF by enhancing neural conduction and reducing neural inflammation.

### 4.1. DOX-induced neuromuscular transmission failure is prevented by exercise preconditioning

Acute respiratory insufficiency, dyspnea and fatigue have been described in patients receiving DOX chemotherapy treatment (Manir et al., 2012; Hirsch et al., 1996). These effects develop both acutely and persist following completion of chemotherapy, with fatigue reported as the most common residual side effect of treatment for survivors of gynecological cancers (Westin et al., 2016). A common contributing factor to these morbidities is deterioration of respiratory muscle function, with the diaphragm being particularly susceptible (Stevens and Sporn, 2014). Increasing evidence in preclinical models demonstrates a reduction in diaphragm muscle specific force production following acute exposure to DOX (Gilliam et al., 2011a; Gilliam et al., 2011b; Doerr et al., 2022; Smuder et al., 1985a; Smuder et al., 1985b); however, the etiology of this deficit is largely unknown. Compromised myofibrillar protein function and impaired calcium uptake are hypothesized to contribute to skeletal muscle contractile dysfunction with evidence suggesting that exercise is sufficient to prevent, at least in part, reductions in myosin, alpha-actinin and SERCA1 protein expression resulting from DOX treatment (Mackay et al., 2021; Kwon, 2020; Guigni et al., 2019). These findings highlight the fact that muscle intrinsic factors likely contribute to DOX-induced diaphragm muscle weakness and also mediate exercise preconditioning protection. However, reduced NMTF observed following prolonged repetitive phrenic nerve stimulation in diaphragm muscles from exercised, DOX-treated rats does indicate that motor neural input also contributes to the beneficial effect of exercise preconditioning to reduce DOX-induced diaphragm dysfunction. Interestingly, protection against NMTF suggests a role for exercise to preserve sarcolemma excitability potentially through the maintenance of synaptic vesicle release, AChR sensitization and/or axonal propagation when the diaphragm is stimulated repeatedly (Davis et al., 2022).

### 4.2. Phrenic axon morphology is preserved by exercise preconditioning

Peripheral neuropathy in response to chemotherapy treatment is a well-established side effect with estimates of persistent symptoms post-treatment ranging from 11% to >80% of patients (Rivera et al., 2018). Clinically, peripheral neuropathy is most commonly identified through surveys and patient reported outcomes, thus the incidence may actually be underestimated due to the focus on overt sensory and motor impairment (Kuroi et al., 2008). A preclinical investigation by Huot et al. on the contribution of neuropathology to chemotherapy-induced muscle weakness utilized both compound muscle action potential responses and motor unit number estimation to demonstrate a loss of motor unit connectivity in the hindlimb muscles of mice treated with cisplatin or FOLFIRI (folinic acid, fluorouracil and irinotecan) (Huot et al., 2021). This work also showed loss of the presynaptic axon terminal of the NMJ. In patients receiving hyperthermic isolated limb perfusion, neurogenic effects of DOX and melphalan were demonstrated in patient biopsies as motor unit changes and fiber type grouping (Bonifati et al., 2000). These data along with our findings support the postulate that presynaptic modifications contribute to reduced muscle strength and fatigue following chemotherapy treatment and highlight that distinct differences exist in comparing specific chemotherapies and muscle types (Bonifati et al., 2000; Huot et al., 2021; Huertas et al., 2020). Specifically, our results demonstrate reductions in phrenic nerve axon and myelin area but show no distinct changes to the NMJ. While future study warrants investigation into whether differences exist at the level of the NMJ between fatigable and fatigue-resistant motor units following acute and chronic DOX myotoxicity, it has been shown that NMTF can develop independent of overt morphological NMJ perturbation (Rowley et al., 2007; Sieck and Prakash, 1995; Fogarty and Sieck, 2023). Under these conditions, it was hypothesized that NMTF resulted from impaired action potential propagation and/or synaptic vesicle recycling. Additionally, distinct alterations to evoked neurotransmission that precede morphological changes at the NMJ may be the result of altered activity of perisynaptic Schwann cells (Martineau et al., 2020). Our results support the notion that NMTF may arise from reduced action potential conduction as the phrenic nerve axons from Sed-DOX rats had a significantly reduced myelin area compared to all other groups. Conversely, exercise training has been shown to promote beneficial adaptations to peripheral nerve structures including axonal growth and remyelination in both healthy skeletal muscles and those exhibiting peripheral neuropathy (Pratt et al., 2021; Maugeri et al., 2021; Udina et al., 2011). This parallels our finding that myelin area is increased in the Ex-DOX group compared to Sed-DOX. Therefore, exercise preconditioning may confer protection against DOX-induced phrenic nerve dysfunction by preventing demyelination.

### 4.3. Exercise preconditioning reduces circulating myokines

Inflammation is a hallmark of DOX toxicity, with reports indicating that exercise is sufficient to reduce its effects within skeletal muscle (Osama et al., 2024; Nunes et al., 2023). However, systemic inflammation originating from the nervous system may be a key component promoting NMTF following DOX treatment. Specifically, astrocytes and microglia are primary mediators of inflammation in the central nervous system (CNS) and neuroinflammation is recognized as a contributing factor to neurotoxicity and skeletal muscle weakness (Ji et al., 2022). SPARC, fractalkine, FSTL-1 and EPO are expressed within the CNS that can be upregulated as part of an inflammatory response (McGovern et al., 2021; Pawelec et al., 2020; Cheng et al., 2017; Bond and Rex, 2014), and which were increased in the plasma of Sed-DOX rats. These myokines have auto- and paracrine effects and while their function does appear to vary depending on the stimulus, each can be increased in neurotoxic conditions (McGovern et al., 2021; Pawelec et al., 2020; Cheng et al., 2017; Bond and Rex, 2014). While these may not all directly increase pathological signaling, their increased circulating levels represent a response to neuropathology (i.e., neuroinflammation) that could contribute to neuromuscular and skeletal muscle dysfunction. Importantly, exercise preconditioning reduced the levels of each of these proteins potentially indicating a lack of heightened neuroinflammatory conditions.

## 5. Conclusions

DOX stimulates the development of diaphragm muscle weakness and fatigue acutely following administration, which is largely prevented by endurance exercise preconditioning. While direct muscle damage has been hypothesized as the primary contributing factor, this study demonstrates for the first time that DOX-induced respiratory insufficiency is the result of impairment in diaphragm muscle contractility and presynaptic signal conduction. We provide functional evidence that acute exposure to DOX causes NMTF, reduced myelin area and increased expression of circulating chemokines. These adverse effects were reduced with preconditioning exercise and these findings provide support for future studies targeting the neuromuscular system as a mechanism to prevent the chronic development of DOX respiratory dysfunction.

## Figures and Tables

**Fig. 4.**
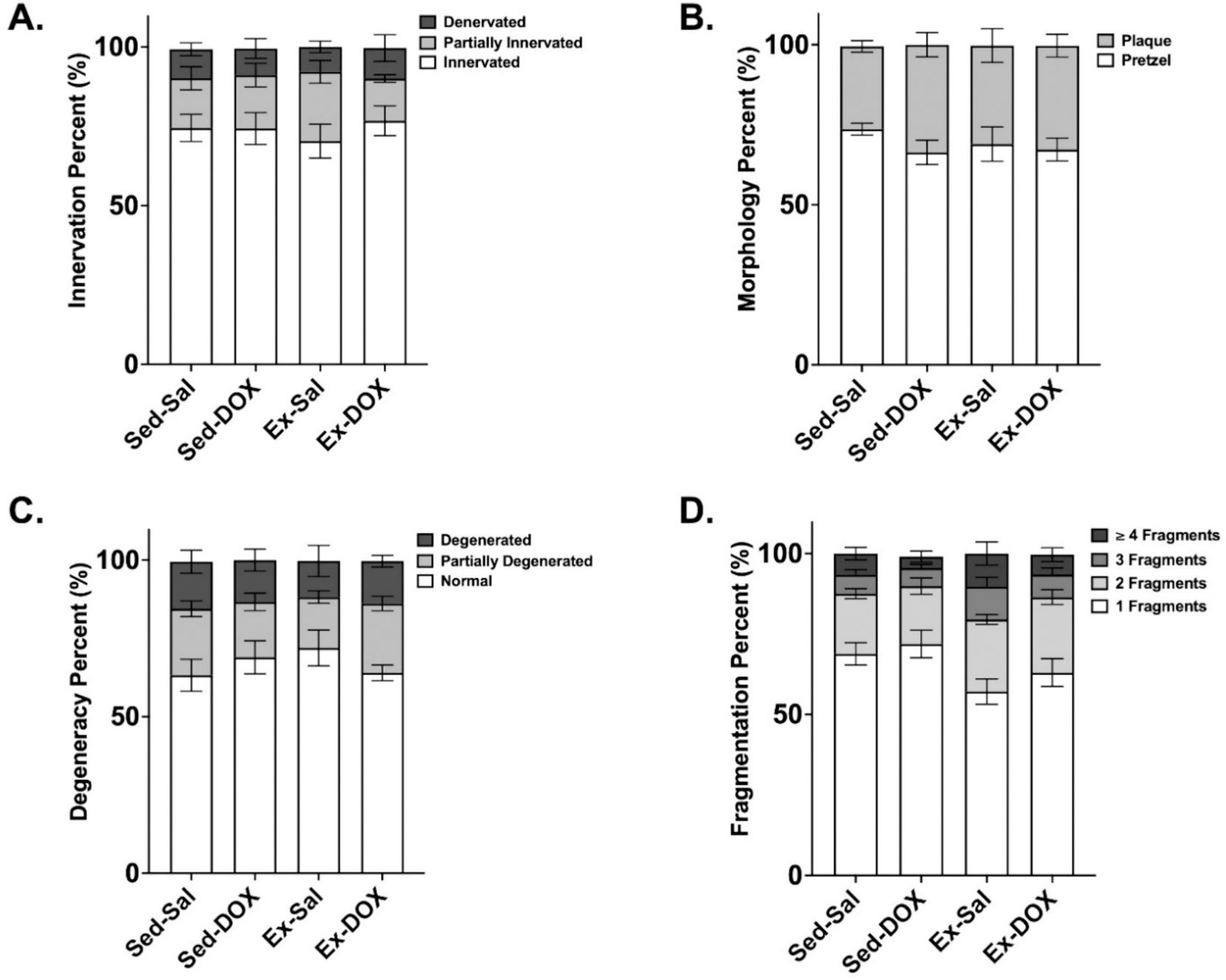

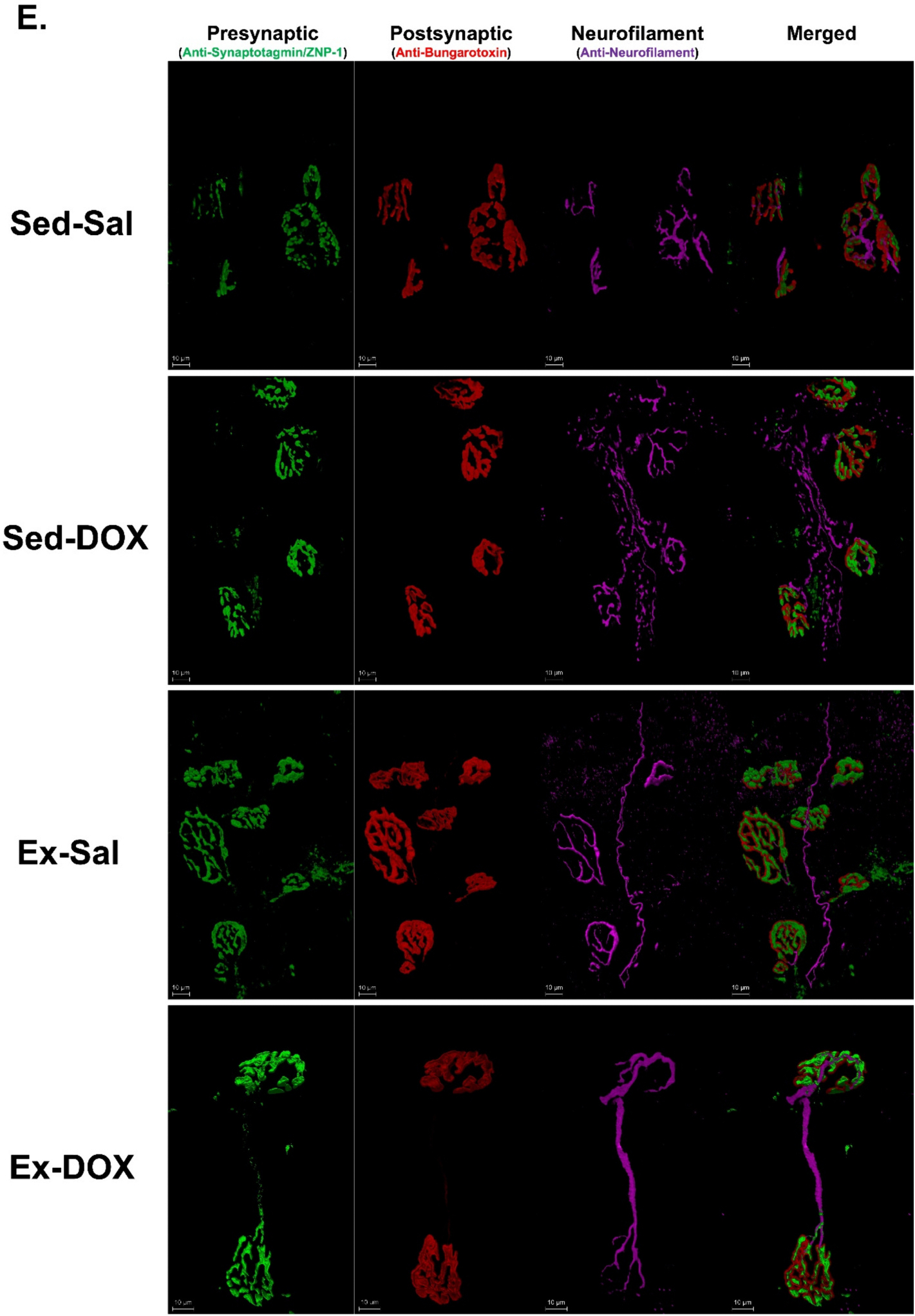
(A-E). Diaphragm neuromuscular junction morphology. A) Percentage of diaphragm NMJs innervated, partially innervated or denervated. B) Percentage of AchR staining exhibiting plaque or pretzel-like post-synaptic morphology. C) Percentage of degenerated post-synaptic NMJ assembly categorized as NMJ normal, partially degenerated or degenerated. D) Diaphragm post-synaptic fragmentation analysis for percentage of AchR staining in 1, 2, 3 or ≥ 4 isolated fragments. Representative images for each condition are shown in Fig. 4E (10–25 NMJs analyzed per animal; *n*=8–10/group). Statistical analysis was completed by two-way ANOVA and Tukey’s multiple comparisons test. Graphs represent mean ± SE. Scale bar represents 10 μm.

**Table 1:** Total body weight at time of treatment and endpoint.

	Sed-Sal	Sed-DOX	Ex-Sal	Ex-DOX

Treatment Weight (g)	265.6 ± 5.80	271.4 ± 5.58	263.5 ± 4.58	271.8 ± 4.72
Endpoint Weight (g)	265.0 ± 5.17	257.6 ± 4.69	271.9 ± 4.22	263.3 ± 4.72
Weight Change (g)	− 0.56 ± 2.06	−15.33 ± 5.37^#^	+8.40 ± 2.54^∨^	− 8.50 ± 4.15

Weight change = endpoint - treatment. Data are presented in grams. Statistical analysis was completed by two-way ANOVA and Tukey’s multiple comparisons test. Data are presented as mean ± SE. *n* = 9–10/group.

# = significantly different vs Sed-Sal and Ex-Sal.

∨ = significantly different vs Ex-Sal.

## Data Availability

Data will be made available on request.

## Abbreviations:

DOX: Doxorubicin
NMTF: Neuromuscular Transmission Failure
NMJ: Neuromuscular Junction
Sed-Sal: Sedentary-Saline
Sed-DOX: Sedentary-Doxorubicin
Ex-Sal: Exercise-Saline
Ex-DOX: Exercise-Doxorubicin
I.P.: Intraperitoneal
mA: amperes
NFinit: Initial Phrenic Nerve Force
MFinit: Initial Direct Muscle Force
ZNP-1: Synaptotagmin-1
NFH: Neurofilament
AChR: acetylcholine receptor
BDNF: Brain-derived Neurotrophic Factor
EPO: Erythropoietin
FSTL-1: Follistatin-1
MSTN/GDF8: Myostatin
SPARC: Secreted Protein Acidic and Rich in Cysteine
FGF21: Fibroblast Growth Factor 2
SE: Standard Error
FOLFIRI: Folinic Acid Fluorouracil and Irinotecan
CNS: Central Nervous System
